# Integrated Metagenomics and Metatranscriptomics Analyses of Root-Associated Soil from Transgenic Switchgrass

**DOI:** 10.1128/genomeA.00777-14

**Published:** 2014-08-14

**Authors:** Archana Chauhan, Abby Smartt, Jun Wang, Sagar Utturkar, Ashley Frank, Meng Bi, Jiang Liu, Daniel Williams, Tingting Xu, Melanie Eldridge, Andres Arreaza, Alexandra Rogers, Hector Castro Gonzalez, Alice C. Layton, Holly L. Baxter, Mitra Mazarei, Jennifer M. DeBruyn, C. Neal Stewart, Steven D. Brown, Loren J. Hauser, Gary S. Sayler

**Affiliations:** a The Joint Institute for Biological Sciences, University of Tennessee Knoxville & Oak Ridge National Laboratory, Knoxville, Tennessee, USA; b The Center for Environmental Biotechnology, University of Tennessee, Knoxville, Tennessee, USA; c Department of Microbiology, University of Tennessee, Knoxville, Tennessee, USA; d Graduate School of Genome Science and Technology, University of Tennessee, Knoxville, Tennessee, USA; e Department of Chemistry, University of Tennessee, Knoxville, Tennessee, USA; f BioEnergy Science Center (BESC), Oak Ridge National Laboratory, Oak Ridge, Tennessee, USA; g Department of Plant Sciences, University of Tennessee, Knoxville, Tennessee, USA; h Department of Biosystems Engineering & Soil Science, University of Tennessee, Knoxville, Tennessee, USA; i Biosciences Division, Oak Ridge National Laboratory, Oak Ridge, Tennessee, USA

## Abstract

The benefits of using transgenic switchgrass with decreased levels of caffeic acid 3-*O*-methyltransferase (COMT) as biomass feedstock have been clearly demonstrated. However, its effect on the soil microbial community has not been assessed. Here we report metagenomic and metatranscriptomic analyses of root-associated soil from COMT switchgrass compared with nontransgenic counterparts.

## GENOME ANNOUNCEMENT

The use of transgenic crops in agriculture continues to increase worldwide, with uses in energy and environmental applications. One potential largely unexplored effect of growing transgenic plants is the alteration of indigenous soil microbial communities (1, 2). Since soil microorganisms play a key role in the global nutrient cycling and maintenance of soil structure (3), alteration of microbial community diversity or activity might have significant effects on the soil ecology and biogeochemical processes. The results of previous studies of soil microbes associated with transgenic plants used non-sequencing-based methods and were of limited scope (4, 5). In this study, we performed metagenomics and metatranscriptomics investigations of soil microbial communities associated with transgenic switchgrass (*Panicum virgatum* L.), in which the endogenous caffeic acid 3-*O*-methyltransferase (COMT) gene was downregulated (6). The field-grown transgenic plants had altered lignin, higher saccharification, and 50% more biofuel production per hectare when grown in Knoxville, TN, USA (6). In addition, greenhouse-grown transgenic switchgrass yielded more ethanol with the use of consolidated bioprocessing (7).

Soil samples were collected from 12 plots (6 each planted with transgenic and nontransgenic plants) on 20 November 2012 from year 2 of the aforementioned field study (6). Soil cores were collected 15 cm from the plants and composited to a depth of 35 cm from the soil surface. Plant roots were gently removed from bulk soil. Soil clinging to the roots was analyzed further. DNA was extracted using the Fast DNA spin kit (MP Biomedicals, Santa Ana, CA). Total RNA was isolated using an RNA PowerSoil total RNA isolation kit (MO BIO Laboratories, Carlsbad, CA). rRNA depletion was performed using a Ribo-Zero rRNA removal kit (Bacteria) (Epicenter, WI) and/or Nugen ovation technology (NuGEN Technologies, CA). Metagenomic and metatranscriptomics libraries were prepared using an Illumina Nextera DNA library preparation kit and a Truseq RNA v. 2 kit, respectively (Illumina, Inc., CA) and sequenced using the Illumina HiSeq 2000 platform in triplicate (biological samples), yielding ~117 Gb of metagenomic and ~53 Gb of metatranscriptomic data.

Raw sequence reads from 32 metagenomic and 13 metatranscriptomic datasets were submitted to the MG-RAST v. 3.3.7 (8) server for downstream analyses. Shotgun sequences were also assembled using Metavelvet (9) and contigs were uploaded to JGI’s IMG/M (10) and MG-RAST pipelines for annotation and analyses. The GC percentages ranged from 64 ± 2% to 66 ± 5%. Metagenomes consisted of ~97% bacteria, ~1% archaea, and ~1 to 2% eukaryota. The major phyla were *Proteobacteria* (40 to 43%), *Actinobacteria* (16 to 25%), *Acidobacteria* (3 to 8%), *Firmicutes* (6 to 8%), *Chloroflexi* (3 to 5%), *Planctomycetes* (3 to 4%), *Cyanobacteria* (2 to 4%), *Bacteroidetes* (2 to 3%), *Germmatimonadetes* (1%), and *Nitrospirae* (0.2 to 0.8%). Analysis of variance showed no statistical difference in the major phyla between transgenic and nontransgenic plant soils. Relative abundances of major taxonomic groups were similar in the DNA and cDNA libraries.

These datasets provide information on the potential long-term effects of transgenic crops on the soil microbial populations. In addition, the systematic and replicated analyses allow direct comparison between the transgenic and nontransgenic counterparts.

### Nucleotide sequence accession number.

Nucleotide sequences obtained were deposited at the NCBI Sequence Read Archive under the accession number SRP044193.
